# Dysprosium magnesium silicate apatite featuring field and temperature stable slow magnetization relaxation†

**DOI:** 10.1039/d0ra07069f

**Published:** 2020-10-14

**Authors:** Pavel E. Kazin, Mikhail A. Zykin, Lev A. Trusov, Alexander V. Vasiliev, Reinhard K. Kremer, Robert E. Dinnebier, Martin Jansen

**Affiliations:** Department of Chemistry, Lomonosov Moscow State University 119991 Moscow Russia kazin@inorg.chem.msu.ru +7 495 9393440; Institute of General and Inorganic Chemistry RAS (IGIC RAS) 31 Leninsky Ave. 119991 Moscow Russia; Max Planck Institute for Solid State Research Heisenbergstrasse 1 70569 Stuttgart Germany

## Abstract

Dy–Mg silicate Dy_8_Mg_2_(SiO_4_)_6_O_2_ has been prepared by high-temperature solid state reaction. It has an apatite type structure (*P*6_3_/*m*) with the Dy atoms fully occupying the 6h site and being in random distribution with the Mg atoms at the 4f site. The compound reveals dual magnetization relaxation with widely varying contributions from fast (FR) and slow (SR) relaxation paths controlled by field and temperature. The SR path is stabilized by a strong magnetic field, exhibits a weak dependence of relaxation time *τ* on field and temperature, and sustains large *τ* of a few seconds up to a temperature of 40 K and under a field of 50 kOe. The analysis of the electronic structure and comparison with the known Dy-doped phosphate apatites suggests that the Orbach and Raman processes are suppressed.

## Abstract 2

The compound reveals dual magnetization relaxation with largely varying contributions from fast and slow relaxation paths controlled by field and temperature. The relaxation times retain values of a few seconds up to 40 K.

## Graphical abstract

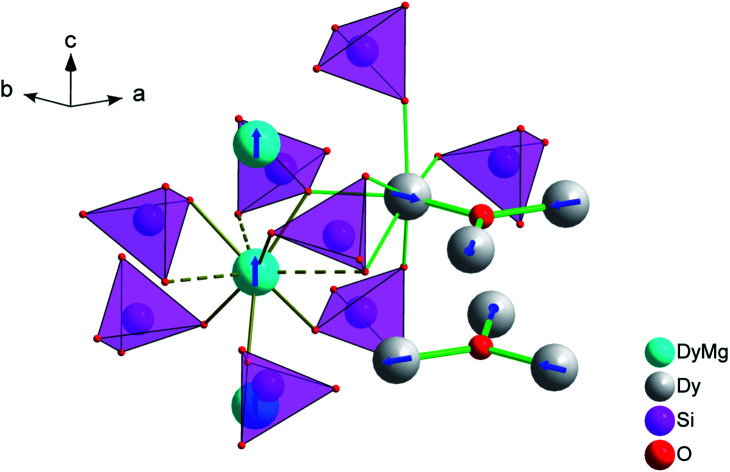

## Introduction

To date hundreds of coordination compounds of open shell d- and f-metals have been shown to exhibit magnetic bistability of a lone metal ion and are referred to as single ion magnets (SIMs) with potential applications in super-dense information recording, molecular electronics, spintronics, and quantum computing.^1–6^ Their key parameters – the energy barrier for magnetization reversal *U*_eff_ and magnetization blocking temperature *T*_b_ (the temperature at which the hysteresis loop closes) – lately have been strongly improved and currently approach 1540 cm^−1^ and 80 K, respectively.^7–10^ Open shell metal ions embedded in a diamagnetic ceramic inorganic matrix constitute a special class of such materials.^11^ Being chemically and thermally much more stable than metal–organic SIMs, such all-inorganic SIMs reach comparably well performing pertinent functional parameters. The most spectacular examples, Dy^3+^-doped phosphate apatites M_10−*x*_Dy_*x*_(PO_4_)_6_(OH_1−_*_x_*_/2_)_2_, where M = Sr, Ca, show *U*_eff_ and *T*_b_ of up to 1043 cm^−1^ and 11 K respectively.^12^ In their crystal lattice, Dy^3+^ occupies the 6h M2 site, has a very short (2.05–2.14 Å) contact to O^2−^ located in the center of the apatite trigonal channel, and forms an effective DyO^+^ cation characterized by a strong axial crystal field and a huge magnetic anisotropy. The full coordination sphere of Dy^3+^ can be considered as a distorted pentagonal bipyramid of oxygen atoms containing the intrachannel oxygen as the bipyramid apex. A very high *U*_eff_ is determined by the Orbach relaxation through the 3^rd^ (M = Ca) or 4^th^ (M = Sr) exited Kramers doublet (KD), the transitions to lower lying KDs being forbidden due to the strict crystal field axial symmetry. Theoretical calculations show that the isolated diatomic unit DyO^+^ has to have a very large barrier for magnetization reversal of 2000 cm^−1^.^13^

Best performance for SIMs is expected and usually found if the metal ions are well separated from each-other in the crystal structure. However there is evidence that spin–spin interactions between the metal cations at certain conditions may slow down magnetization relaxation instead of accelerating it.^14–17^ Presence of such interactions is also important in order to correlate spins of magnetic centers, which is a necessary prerequisite for establishing qubits for quantum computers.^4,5^ Therefore compounds with relatively short distance between paramagnetic centers (however not magnetically long-range ordered) may be of pertinent interest.

Recently we reported on the synthesis and magnetic properties of dysprosium containing calcium silicate apatites: Dy-rich Dy_8_Ca_2_(SiO_4_)_6_O_2_, and diluted Y_7.75_Dy_0.25_Ca_2_(SiO_4_)_6_O_2_.^18^ In the first compound, similar to the phosphates, about three-fourth of the Dy atoms occupy the M2 site so that flat Dy_3_O triangular groups perpendicular to the *c*-axis form. The remaining Dy atoms share the 4f M1 site with Ca. A Dy2–O4 distance of 2.21 Å is longer than that in the respective phosphate, which reduces somewhat the crystal field anisotropy. The latter compounds exhibit fast magnetization relaxation in the absence of an external magnetic field, but with increasing field slow relaxation paths gradually arise. The relaxation is considerably slower in the Dy-rich compound than in the diluted one. Noteworthy, the fraction of slow relaxing magnetization depends strongly on magnetic field and temperature, while the relaxation time – being of an order of magnitude of one second – depends weakly on these parameters. With the aim to further investigate into this peculiar type of slow relaxation and to optimize SIM parameters by increasing the crystal field anisotropy, we have prepared and analyzed an analogous compound with the smaller divalent metal cation Mg – dysprosium magnesium silicate apatite Dy_8_Mg_2_(SiO_4_)_6_O_2_.

## Experimental

Ceramic pellets with the nominal composition Dy_8_Mg_2_(SiO_4_)_6_O_2_ were prepared starting from oxides as follows. Analytical grade (Aldrich) Dy_2_O_3_, MgO, and SiO_2_ were pre-annealed in an electric furnace in air at 1000 °C for 2 h and air quenched in order to remove any water and carbonate to ensure defined weighing forms. The oxides taken in the stoichiometric amounts were ground and mixed in an agate mortar to get *ca.* 2 g of the final compound. The mixture was pressed in pellets (5 × 10^3^ kg cm^−1^), and the pellets were heat treated in an electric furnace in air. The temperature was raised to 1450 °C for 1.5 h and maintained for 24 h. Subsequently, the samples were air quenched. The sintered pellets were reground, pressed in pellets again, and heat treated once more in a similar way. The obtained samples were further heated to 1580 °C for 1.5 h, kept at this temperature for 4 h, and air quenched. As prepared samples were well sintered ceramics of white color with a faint yellow gleam.

X-ray powder diffraction (XRPD) patterns were collected on a D8 Bruker-Advance powder diffractometer with Cu-Kα1 radiation from a primary Ge(111) Johansson-type monochromator in Bragg–Brentano geometry using Lynx-Eye position-sensitive detector. The intensities were collected in the 2*θ* range 10–120° with a step of 0.00657°. The Jana2006 program was used for the crystal structure refinement.^19^ The background was treated using Legendre polynomials. XRPD profile and crystal cell parameters, atomic parameters including isotropic thermal displacement factors, and the Dy–Mg occupancies at the M1 and M2 sites were refined, whereas the chemical composition was fixed to the nominal one.

Measurements of magnetic properties were performed using a Quantum Design SQUID MPMS magnetometer. The rod-like piece of the sample pellet was firmly fixed in a polyethylene straw without any additional material with the rod's long dimension along the magnetic field (a few mg for dc and *ca.* 100 mg for ac measurements). For this purpose we prepared the latter sample with the size larger than the straw diameter, pushed it into the straw with a force, and additionally fixed it making small cuts of the straw just below and above the sample. The smaller sample was tightened in a small compartment of the straw made by appropriate cutting and bending of the straw parts. The field dependence of magnetization was measured at a temperature of 2 K in the field range 0–70 kOe with field sweeping rates of 0.4 and 2 kOe min^−1^. dc susceptibility *χ*(*T*) was measured under a field of 1 and 10 kOe in the temperature range 2–300 K. For the joint analysis with the ac susceptibility data, *χ*(*T*) was measured in the temperature range 2–50 K in fields of 3.5, 4.5, 7, 9, 18, 22, 45 and 55 kOe in order to extract differential susceptibility values. Real *χ*′ and imaginary *χ*′′ parts of ac susceptibility *χ*_ac_ were measured in the temperature range 2–50 K in the ac field frequency range 0.1–1488 Hz with an ac field amplitude of 4 Oe under dc fields of 0, 1.5, 4, 8, 20, and 50 kOe. For the measurements under fields of 20 and 50 kOe, the lowest temperature was limited to 4 and 12 K, respectively, since at the lower temperatures the magnetization was close to saturation and the ac signal was very low and noisy. The ac signal had a sine wave shape without visible distortions, which was also checked by appropriate fitting. This implied that there was no sign of shifting or vibrating of the sample during the ac measurements, which otherwise could affect the measurement results. The core diamagnetic contribution was calculated using Pascal's increments. The demagnetizing factor of the sample was taken into account to correct the internal magnetic field for the *M*(*H*) data and susceptibility for the *χ*(*T*) data. The sample shape was considered approximately as a cylinder with a length-to-diameter ratio of 2.5.

The modeling of crystal field parameters, electronic structure, susceptibility, and magnetization was conducted using the PHI^20^ and CONCORD^21,22^ programs. A primary analysis of the crystal field at Dy^3+^ was performed using a point-charge model. The ac susceptibility data were further corrected comparing them with the differential dc susceptibility data, both taken at *T* = 50 K, when all paramagnetic centers relax very fast. Performing ac measurements at relatively high dc fields (above 4 kOe) for different compounds we regularly observed a very slow (hours) relaxation process in the MPMS magnetometer, which might be connected to a tiny creep of the dc field. It causes the drift of the ac susceptibility baseline especially at low frequencies. It is effectively expressed in the negative phase shift of the ac signal. The shift is roughly inversely proportional to the ac field frequency *f* and can be distinguished measuring fast relaxing paramagnets (*e.g.* Dy_8_Mg_2_(SiO_4_)_6_O_2_ at 50–80 K and CuSO_4_·5H_2_O at lower temperatures). In several cases, the ac data were corrected for this effect by refining a single shift parameter for the *χ*_ac_(*f*) set with the phase shift being proportional to 1/*f*. The correction was substantial for the points measured at 12 K, 50 kOe; 4 K, 20 kOe; and 2 K, 8 kOe resulting in an increased standard deviation values for estimated parameters. The correction was not needed if the measurements were conducted later than approximately 1 day after the last setting of magnetic field.

The magnetization relaxation processes were analyzed by simultaneous fits of the frequency (*ω* = 2π*f*) dependence of *χ*′ and *χ*′′ in the generalized Debye model,^23^eqn (1) and (2).1
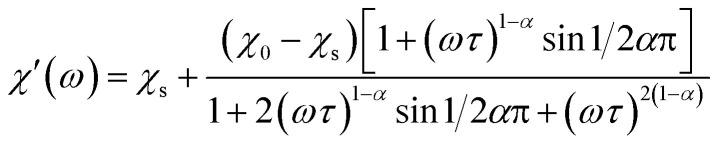
2
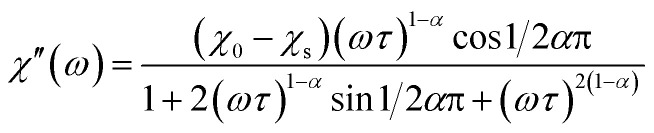


The relaxation time *τ*, relaxation time distribution width *α*, and contribution of slow relaxing centers Δ*χ* = *χ*_0_ − *χ*_s_ to the total susceptibility *χ*_0_ were determined. When two relaxation processes were assessed a second term was added to eqn (1) and (2) with respective contribution Δ*χ*_1_. The analysis of a low-frequency relaxation was performed taking *χ*_0_ as a fixed value equal to a differential susceptibility value obtained from corresponding dc susceptibility measurement data. This allowed stable convergence of the refinement even when an expected maximum of *χ*′′(*f*) was at a frequency of an order of magnitude lower than the lower boundary (0.1 Hz) of the ac field frequency range used in the measurements. The reliability of this approach we have checked by the analysis of our earlier *χ*_ac_(*f*) data with well developed *χ*′′(*f*) peaks reported in ref. 12. *α* was normally refined for low temperatures and subsequently fixed to the average low temperature value to estimate relaxation parameters at higher temperatures.

## Results and discussion

### Crystal structure details

**Fig. 1:**
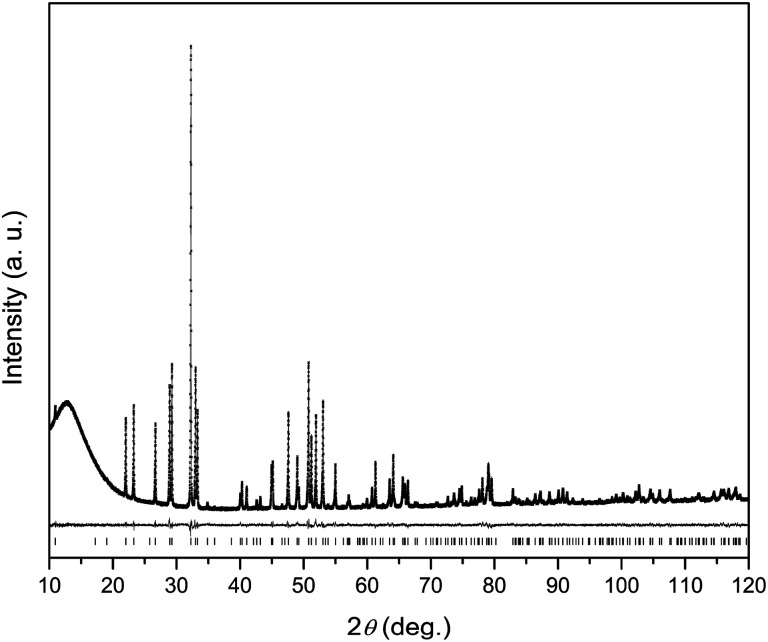
X-ray powder diffraction pattern of Dy_8_Mg_2_(SiO_4_)_6_O_2_. Observed (crosses), calculated (solid line) and difference (solid line below) plots. Positions of Bragg reflections are shown as strokes underneath.

**Fig. 2:**
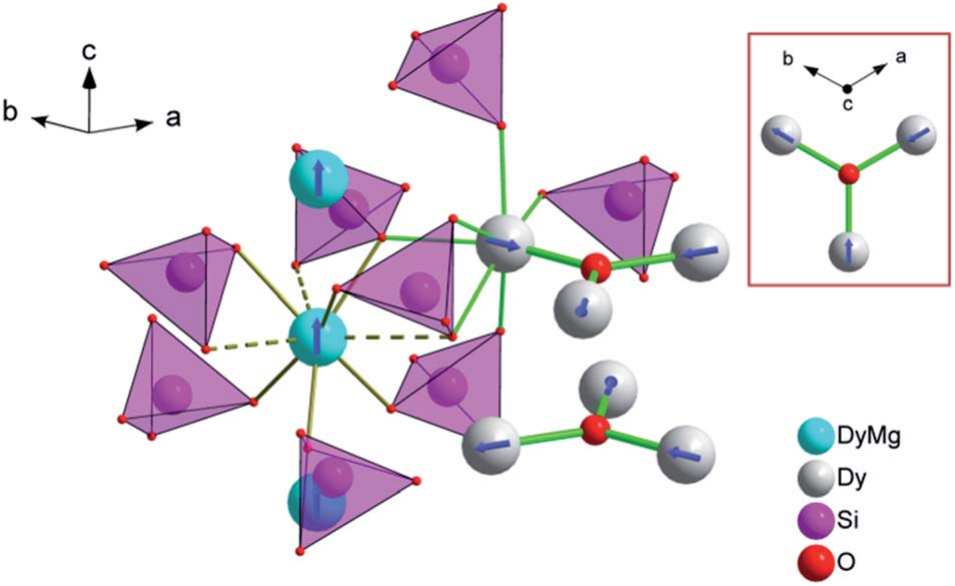
A fragment of the crystal structure of Dy_8_Mg_2_(SiO_4_)_6_O_2_. The M1 site atoms (Mg, Dy) are represented by cyan spheres, the M2 site atoms (Dy) – by grey spheres. The tetrahedrons depict SiO_4_^4−^ anions. The coordination environment is shown only for one metal ion at the M1 and one at the M2 site. M1 site: solid olive lines – shorter connections to three O1 (lower) and to three O2 (upper) atoms, dashed lines – longer connections to three O3 atoms. M2 site: thick green line – connection to O4 (intra-channel O^2−^), thin green lines – connections to other oxygen atoms belonging to orthosilicate-groups. An arrangement of the M1 and M2 atoms in the crystal lattice is shown. The M2 atoms (Dy2) form flat triangles (M2)_3_(O4) stacked along *c*-axis so that a trigonal channel arises with the “walls” of the M2 atoms building face-shared (M2)_6_ octahedrons running along the *c*-axis. The M1 atoms form linear rows along the *c*-axis in-between the channels. The easy magnetization axes are shown for Dy^3+^ ions at the M1 and M2 sites. The depicted mutual orientation of easy axes at the M2 sites corresponds to a minimum of inter-ion dipolar energy. In the rectangular inset, the (M2)_3_(O4) triangle is shown as the projection along *c* axis to clarify directions of the easy magnetization axes.

The XRPD pattern of the Dy_8_Mg_2_(SiO_4_)_6_O_2_ sample is shown in Fig. 1. The broad peak at low *θ* is connected to the X-ray scattering from the sample holder. All Bragg reflections correspond to the apatite structure with the *P*6_3_/*m* space group. No crystalline impurity phases were detected down to 0.2% of the maximum line intensity. The unit cell parameters are *a* = 9.3081(2) Å, *c* = 6.6742(2) Å. In reference to Dy_8_Ca_2_(SiO_4_)_6_O_2_,^18^*a*-axis is only slightly smaller, by 0.6%, while *c*-axis shrinks by 2.1%. The reduced cell parameters are apparently due to the smaller size of Mg^2+^ in comparison with Ca^2+^. The crystal structure data are summarized in Tables S1–S3.† A fragment of crystal structure is shown in Fig. 2.

In the apatite-type crystal structure, 4 metal ions are located at the M1 site and 6 at the M2 site.^24^ M1 is coordinated by 6 oxygen atoms of the silicate group forming slightly twisted prism complimented by three longer M1–O contacts through the prism side faces (C_3_ site symmetry). M2 is bonded to one intrachannel O^2−^ (O4) and to 6 oxygen atoms belonging to silicate groups thus forming a distorted pentagonal bipyramid (C_s_ site symmetry). Similar to the Dy–Ca silicate, Dy prefers the M2 site while it shares the M1 site with the divalent cation. However, compared to the Dy–Ca compound the Dy preference to the M2 site is higher, the site occupancies being 97% *vs.* 94%. And the compound can be represented by the explicit formula (Dy_0.972_Mg_0.028_)_6_(Dy_0.542_Mg_0.458_)_4_(SiO_4_)_6_O_2_.

We now will consider the coordination spheres of Dy^3+^ at the M1 and M2 sites in the prepared compound in comparison with the Dy–Ca silicate apatite. M1 site: on moving from the Ca- to Mg-containing silicate, the distances to the 6 closest oxygen atoms significantly decrease, from 2.34 to 2.29 Å to O1 and from 2.41 to 2.34 Å to O2. This shortening of the average distance is quite expected, as this site is equally occupied by Dy^3+^ and Mg^2+^, and the latter is smaller than Ca^2+^. The distance to the remote oxygen atoms (O3) remains nearly the same, 2.80–2.81 Å. Consequently one would expect an increase of the crystal field strength at M1 site. M2 site: the closest distance is to intrachannel O^2−^ (Dy2–O4, apex of the pentagonal bipyramid). It decreases from 2.21 to 2.18 Å on going from the Ca- to Mg-based silicate. As this oxygen atom provides the largest contribution to crystal field anisotropy^18^ we expect that the magnetic anisotropy in the Dy–Mg compound at the M2 site is also higher. It is interesting to note, that at the same time the distance to an opposite apex of the bipyramid (O2) increases from 2.37 to 2.38 Å – a kind of trans-effect. Since O2 also contacts the metal cation at the M1 site, the enlargement can be explained by a smaller size of Mg^2+^ forming a shorter and stronger connection to O2. Distances to coordinated oxygen atoms in the bipyramid base are 2.29 (2 × O3), 2.44 (2 × O3), 2.68 (O1) and 2.27, 2.42, 2.74 Å in the Ca- and Mg-based compounds, respectively, and would have a comparable effect on the crystal field.

### dc susceptibility and magnetization

**Fig. 3:**
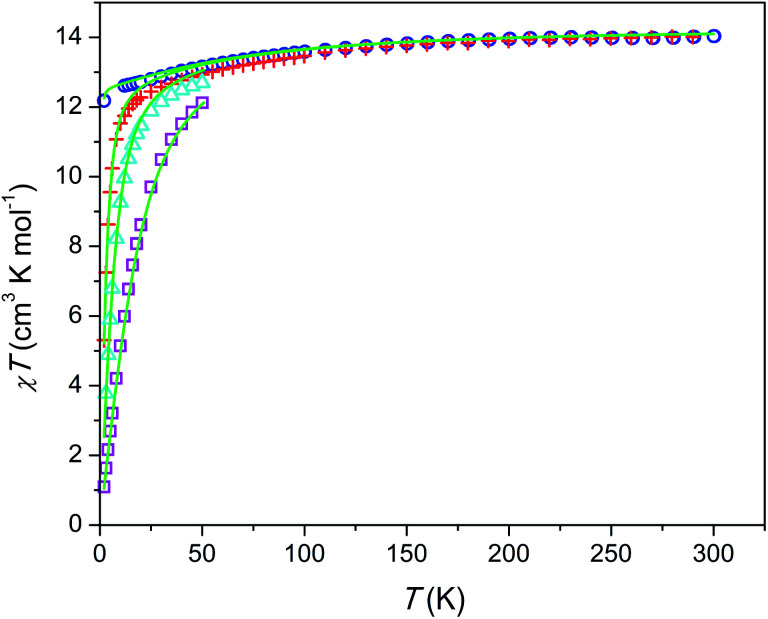
Temperature dependence of *χT* product per mol of Dy at different magnetic fields. Blue circles, *H* = 1 kOe; red plus signs, *H* = 10 kOe; cyan triangles, *H* = 22 kOe, magenta squares, *H* = 55 kOe; green lines, modeling (see text).

The temperature dependence of *χT* product is shown in Fig. 3. The magnetic susceptibility was modeled as a powder average value as follows. The Hamiltonian included spin–orbit coupling, crystal field interaction, and Zeeman effect over the whole |*L*, *M*_L_, *S*, *M*_S_〉 basis for the ^6^H term of Dy^3+^ as implemented in the PHI program.^20^ Using the experimentally obtained atomic coordinates in the crystal structure, all crystal field parameters at the M1 and M2 sites were being repeatedly calculated at varying partial charges at oxygen atoms. The partial charges of oxygen atoms of the SiO_4_^4−^ anion were constrained to be equal and the partial charge at the intrachannel O^2−^ anion (O4) was constrained to be a factor of 2 larger. Hence only one parameter (the partial charge) was varied to fit the magnetic susceptibility. The obtained crystal field parameters and energy levels for Dy^3+^ at the M1 and M2 sites are listed in Tables S4 and S5.†

At high temperatures, *χT* approaches a theoretical free ion value of 14.2 cm^3^ K mol^−1^. At low temperature and low field limits, *χT* is close to 12.5 cm^3^ K mol^−1^. This value corresponds to the ground KD with *M*_J_ = ±15/2. According to the modeling, the first exited KD corresponds to *M*_J_ = ±13/2 and lies at 89 and 174 cm^−1^ for the M1 and M2 sites, respectively. In comparison with the Dy–Ca silicate (64 and 168 cm^−1^, respectively) the magnetic anisotropy enhances substantially at the M1 site and slightly at the M2 site. The directions of easy magnetization axes are shown in Fig. 2. Dy2 has an easy magnetization almost coinciding with the Dy2–O4 bond direction (inclined to it only by 2.7 deg. in the *ab* plain). In the Dy_3_O triangle these axes are found at the angle of 120 deg. to each other. Dy1 has an easy magnetization axis parallel to the *c*-axis, so that its axis is perpendicular to the Dy2 easy axis.

**Fig. 4:**
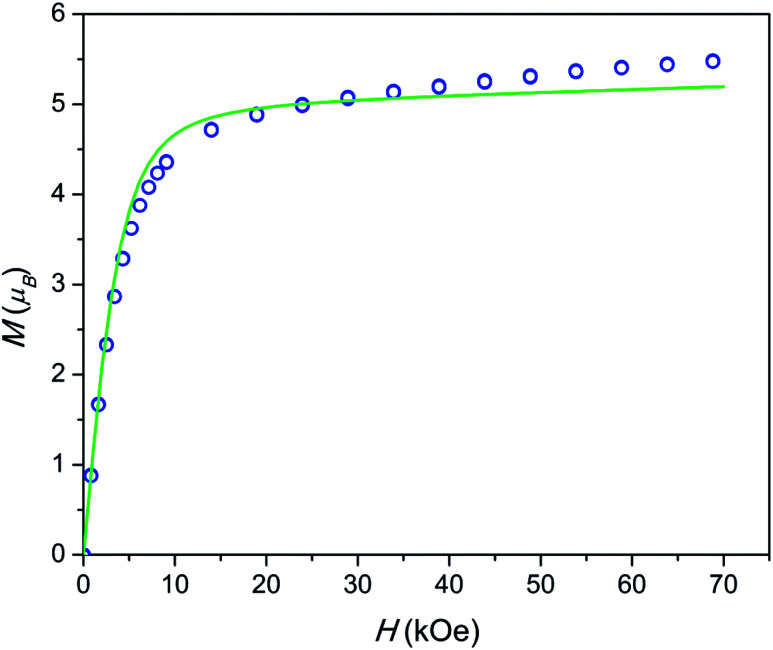
Field dependence of magnetization at a temperature of 2 K. Blue circles – experimental points, green line – modeling.

The field dependence of magnetization is shown in Fig. 4. The magnetization is reversible within the timescale of the measurements. This limits the characteristic maximum time *τ* of a possible slow relaxation to approximately 10^2^ seconds. The experimental points fit well to the modeled curve at low fields. However, at intermediate fields the magnetization is slightly overestimated, while at high fields, just in opposite, underestimated. We can also see that *χT*(*T*) at the highest and the lowest field fits well to the model (see Fig. 3), while at intermediate fields it is slightly overestimated. The exact reason for it is not clear yet. Similar deviations in *M*(*H*) we have observed for the Dy–Ca silicate apatite and we have assumed as possible reasons inter-ion dipole or/and exchange interactions.^18^ Here we performed a tentative analysis of the dipolar interactions restricting them to the (Dy2)_6_ octahedron. Dipolar energy for every pair of Dy^3+^ ions was calculated using formula (3).3
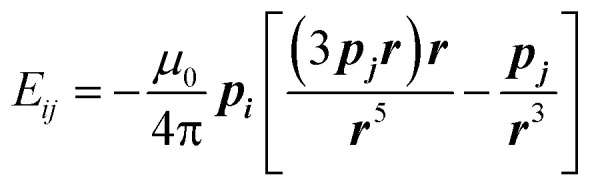
where ***p***_***i***_ and ***p***_***j***_ are the vectors of the Dy^3+^ magnetic moments (10*μ*_B_) and ***r*** is the radius-vector.

The analysis resulted in a complicated 8-levels energy system spreading over ∼10 cm^−1^ (see Fig. S1†). If we take into account Dy1 atoms and remote Dy2 atoms and possible exchange interactions, it will entangle the situation even more. The average dipolar energy per one Dy^3+^ ion is of 1.7 cm^−1^ only. It is comparable with the Zeeman splitting in the Dy^3+^ ground doublet under a field as small as 1.8 kOe. In principle, the increasing magnetic field may bring to the local energy minimum sequentially states with differing magnetic moments. This may cause the observed deviation from the model.

### Relaxation of magnetization

The frequency dependence of ac susceptibilities is shown in Fig. S2–S7.† Without external dc field the magnetization relaxation is fast (denoted as FR) and is revealed as the onsets of the *χ*′ drop and the *χ*′′ increase at higher frequencies. Under a field of 1.5 kOe a weak lower frequency growth is detected on both, *χ*′ and *χ*′′. It becomes pronounced at 3–8 K and a small peak on *χ*′′ can be observed above 5 K. This feature corresponds to an arising fraction of a slow relaxation path (denoted as SR). The major part of the magnetization continues to relax fast. At higher fields the SR fraction becomes larger (to the extent of FR fraction) and the *χ*′′ peak shifts to lower frequencies and goes out of the frequency range investigated.

For the FR path, we can estimate *τ* only tentatively. If we assume that the fraction of susceptibility remaining after the SR path relaxes with one characteristic *τ*, an estimated *τ* value (under zero and 1.5 kOe field) is of an order of a microsecond.

**Fig. 5:**
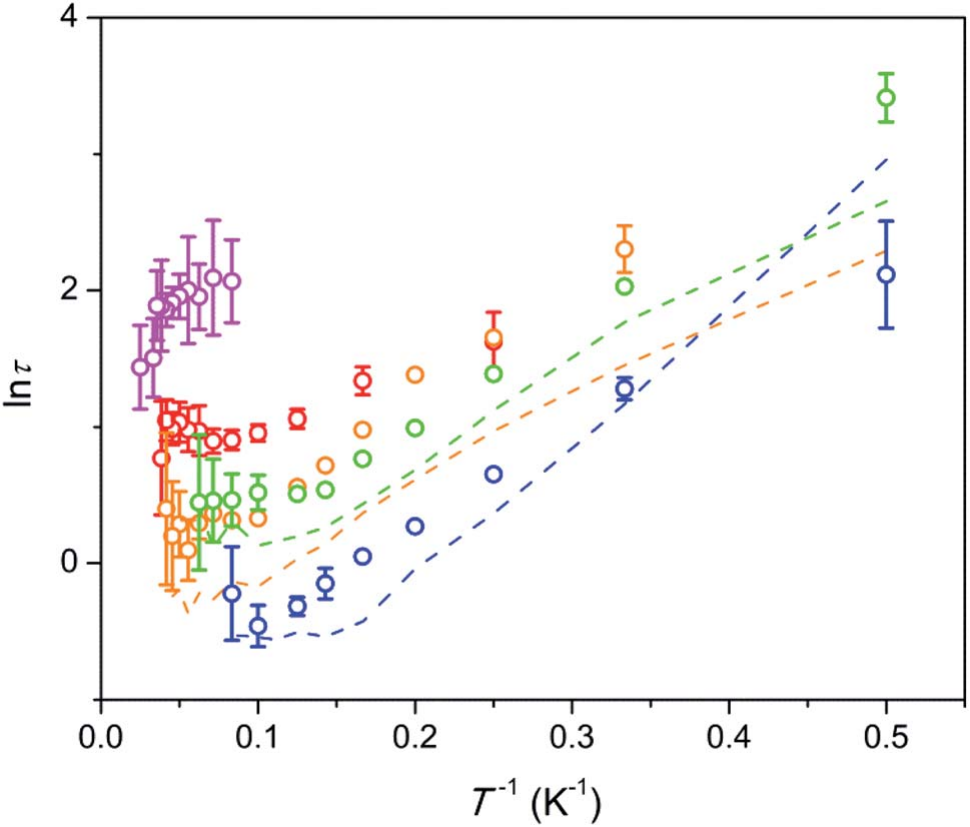
Dependence of ln *τ* on inverse temperature for the SR path. Symbols – data for the Dy–Mg silicate apatite; lines – data for the sister compound, Dy–Ca silicate apatite.^18^ Error bars correspond to two standard deviation values. They are shown if exceeding the symbol size. Blue, green, orange, red, and magenta – under a field of 1.5, 4, 8, 20, and 50 kOe, respectively.

**Fig. 6:**
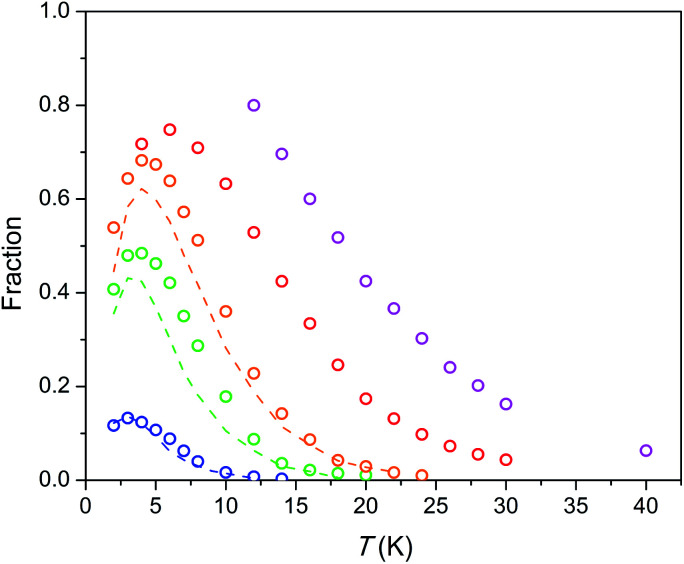
Fraction of susceptibility which relaxes slowly (by the SR path) *vs.* temperature under different dc fields. Symbols – data for the Dy–Mg silicate apatite, dashed lines – data for the Dy–Ca silicate apatite.^17^ The color designates an applied magnetic field as in Fig. 5. Error bars do not exceed the symbol size.

The temperature dependence of the relaxation times *τ* estimated for the SR path is presented in Fig. 5. Fractions of susceptibility (*F*) involved in the SR path under different magnetic fields are shown in Fig. 6 as functions of temperature. The relaxation behavior resembles that in the Dy–Ca silicate apatite^18^ which was analyzed in a narrower field-temperature range though. For the sake of comparison the respective data for the latter compound are also shown in Fig. 5 and 6.

The relaxation is commonly considered as having contributions from four processes: quantum tunneling of magnetization (QTM), direct process (under magnetic field only), Raman, and Orbach ones.^25,26^ All the processes except QTM are temperature dependent.

For the SR path the *τ* values span from 0.4 to 30 s in the whole field (1.5–50 kOe) and temperature (2–40 K) ranges studied. The Dy–Mg compound shows slightly larger *τ* values of SR in comparison with the Dy–Ca compound.^18^ Such behavior may be expected since the crystal lattice is compressed by the Mg for Ca substitution. However the changes of the SR parameters are surprisingly small. This implies the SR effect to be robust toward the changes in the crystal lattice.

In general, *τ* of SR decreases about one order of magnitude with increasing temperature and then stabilizes at high temperatures. With increasing dc field the SR slows down by about one order of magnitude. In the analysis of the field dependence of *τ*, two concurrent processes are considered commonly, QTM and direct ones. QTM restricts *τ* at low fields while direct process acts at higher fields. Usually a maximum of *τ* is observed under an intermediate field of a few kOe whereas under high fields, *τ* is expected to be inversely proportional to H^4^.^26,27^ We do not see such behavior. Instead, we observe two *τ* values from two paths with largely varying contributions. Obviously, the same paramagnetic center may relax fast or slow depending on the field and the temperature applied. In ref. 18 we offered a phenomenological explanation for observing several *τ* values from the single paramagnetic center. Two requirements have to be fulfilled. First, the relaxation is determined mostly by the component of magnetic field vector parallel to the easy magnetization axis of the paramagnetic center. And second, the easy magnetization axes are randomly oriented in the sample (due to random orientation of grains in ceramics or powder). Then, if *τ* is small under low fields, then jumps fast in a narrow field range, and changes weakly under higher fields, we will observe two peaks on *χ*′′, corresponding to two values, *τ*_1_ and *τ*_2_ characterizing low and high field relaxations respectively. For such a jump we may introduce an effective threshold field *H*_t_, above which the relaxation slows down sharply.

In our case we may assume that under a low field a fast QTM channel exists and the magnetization relaxes by the FR path only. With increasing magnetic field the relaxation time increases with acceleration (in accord with *τ* = *a* + *bH*^2^, where *a* and *b* are constants).^26^*a* and *bH*^2^ become equal at a certain field. This field can be considered as the mentioned above effective threshold field *H*_t_ above which we start to observe the SR contribution. In order to be consistent with the model of ref. 18 and experimental data the fast growth of *τ* with *H* has to slow down at higher fields. That we observe indeed for the SR path. Importantly, *τ* of the SR path continues to grow constantly with the field up to the highest field applied. This tendency is valid for the whole temperature range studied. At these conditions one may expect a direct process to contribute considerably to the SR. Besides it, a specific Raman II process may prevail at high fields in a wide range of temperatures.^28^ However both processes are characterized by a fast drop of *τ* with the field (for Raman II, *τ* is approximately inversely proportional to *H*^2^). Apparently the observed constant growth of *τ* with the field cannot be attributed either to the direct or Raman II processes. Taking into account weak temperature dependence of *τ* we may also rule out any meaningful contribution from Raman I (field independent) and Orbach processes. Some results of the modeling of *τ* values considering the discussed mechanisms are shown in Fig. S8.† Apparently no good fitting is possible for the observed temperature-field dependence of *τ*. This allows us to assume that the relaxation in the regime of high field is connected to an unknown process which we may call an FTW process (FTW – field and temperature weakly dependent). Noteworthy, we do not observe an appreciable decrease of *τ* even at the highest temperatures at which Raman and Orbach processes have to cause fast drop of *τ* with the increasing temperature. Hence the FTW process seems to persist as the major one in the whole temperature range used.

Multiple relaxation is a quite common phenomenon for SMMs and SIMs. In a number of cases it was attributed to the same paramagnetic center.^29^ And there are few examples of complexes of Co^2+^,^30–34^ Ni^2+^,^35^ and Cu^2+^,^36^ where the slower relaxation path shows weak temperature dependence like in our case. A slow decrease with increasing temperature may even turn back to a weak increase of *τ* at higher temperatures.^36^ Such relaxation path disappears on dilution and is assumed to be supported by inter-ion interactions. In our recent work we showed a similar behavior of the SR path for Tb^3+^ in Ca_10−*x*_Tb_*x*_(PO_4_)_6_(OH_1−*x*/2−*δ*_)_2_ which persisted in the compound with *x* = 0.5 and was absent in diluted one with *x* = 0.1.^29^ As was discussed above, the SR path for Dy_8_Ca_2_(SiO_4_)_6_O_2_ is similar to that observed in the present study. The dilution (composition Y_7.75_Dy_0.25_Ca_2_(SiO_4_)_6_O_2_) does not cancel the SR path, but results in orders of magnitude shorter *τ* as well as in the modification of the temperature dependence of *τ*, which becomes close to that commonly observed: accelerated decrease of ln *τ* with decreasing of *T*^−1^.

Here for Dy_8_Mg_2_(SiO_4_)_6_O_2_ we observe the FTW relaxation in considerably wider ranges of temperature and magnetic field. Therefore it is worthwhile to analyze other possible relaxation processes in order to understand at which conditions the FTW process can screen them.

An Orbach process implements magnetic moment reversal through the excitation to a higher energy KD and determines an Arrhenius type temperature dependence of *τ* according to the equation *τ* = *τ*_0_ exp(*U*_eff_/*kT*).^26^ At the most anisotropic M2 site in Dy_8_Mg_2_(SiO_4_)_6_O_2_, the ground multiplet ^6^H_15/2_ splits into eight KDs with the highest one at 587 cm^−1^ above the ground KD (see Table S5†). The transverse *g*-factors and probability of the magnetization reversal become large for the 5^th^ exited KD found at 517 cm^−1^. Therefore *U*_eff_ cannot exceed the latter number. In the analogous Dy-doped calcium phosphate apatite the Orbach process was characterized by *U*_eff_ = 790 cm^−1^ and *τ*_0_ = 10^−12^ s.^12^ Taking a measured *τ* value of 4.2 s at the highest *T* of 40 K (under a field of 50 kOe) and assuming *τ*_0_ = 10^−12^ s we derive *U*_eff_ to be higher than 872 cm^−1^. This value is considerably larger than the estimated energy of the 5^th^ exited KD. It is possible that the probability of the transition is diminished due to some factors, *e.g.* a kind of inter-ion interaction and (or) strong magnetic field. Then, alternatively, taking the same *τ* and *U*_eff_ = 517 cm^−1^ we obtain that *τ*_0_ has to exceed 3.5 × 10^−8^ s. This value is out of the range of the values obtained for high-energy-barrier SIMs 10^−12^ to 10^−10^ s.^2,7–10,12^ Hence the expected Orbach process is apparently suppressed.

In the Dy-doped phosphate apatites studied earlier in the intermediate temperature range 30–40 K, the relaxation rate was mostly determined by a Raman process.^12^*τ* values were below 0.1 s both under zero and non zero (4 kOe) fields. In the Dy–Mg silicate apatite observed *τ* values are more than one order of magnitude larger while expected *U*_eff_ is a factor of 1.5–2 smaller. Apparently the Raman process is hindered in reference to that in the Dy-doped phosphate apatites.

Very recently the suppression of the Raman process was proposed to guide the relaxation of magnetic moment of a Ho lone atom attached to the MgO surface.^37^ Whereas under low magnetic field the relaxation was fast and thermally activated, under a field of 80 kOe it remained very slow exceeding 10^3^ s at temperatures up to 30 K. It was rationalized by DFT calculations considering a Raman process involving local molecular vibrations.

The field-temperature dependence of the SR fraction of Dy_8_Mg_2_(SiO_4_)_6_O_2_ (Fig. 6) resembles that in the Dy–Ca silicate apatite,^18^ however with slightly higher *F* values. As the main source of SR, we proposed Dy^3+^ in the M2 site as having much stronger anisotropy.^18^ Then the increased *F* may be attributed to higher occupancy of the M2 site by Dy. The SR fraction grows uniformly with increasing field in the whole temperature range. Obviously, magnetic field stabilizes species responsible for the SR path while thermal energy destroys them. The existence of a maximum in *F*(*T*) was also considered in our previous work.^18^ It may be connected to the different magnetic response from paramagnetic centers oriented differently with their easy magnetization axis toward magnetic field so that the slow relaxing species may be under a field component *H*_*z*_ large enough to approach magnetization saturation. It should be mentioned that if only the Dy2 atoms are involved in SR, the maximum fraction of SR cannot substantially exceed 0.75, as the Dy1 atoms contribute only to FR. Under a field of 50 kOe we observe higher values, and the maximum has not been achieved. Hence it cannot be excluded that the Dy1 atoms are also involved in SR at certain fields and temperatures.

**Fig. 7:**
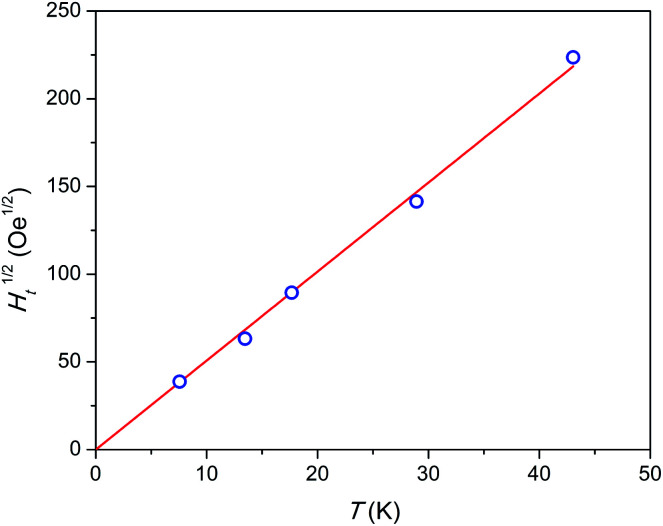
Temperature dependence of square root of threshold field *H*_t_. Blue symbols – experimental points, red line – linear fit. The highest temperature point was estimated by extrapolation.

Admitting the mechanism of the multiple relaxation proposed in ref. 18 the temperature dependence of effective threshold field (*H*_t_) can be estimated. As an approximate value we may take the applied field at a small enough *F* value of 0.05 measured with a reasonable accuracy. The results are shown in Fig. 7. *H*_t_(*T*) is not linear, but rather follows a linear *H*_t_^1/2^(*T*) behavior, *i.e. H*_t_ is proportional to temperature in power of 2. This result appears surprising since the energy (Zeeman splitting and thermal energy) supplied to the system by both factors has to be proportional to their value. And one may expect a linear function. Neither the nature of these SR species nor the reason for the *H*_t_(*T*) behavior observed is clear yet. We may assume that collective effects are involved coupling several or all Dy^3+^ ions to resist fast relaxation. Additionally, complex inter-ion interactions may produce an energy level structure such that, on increasing the energy of an electronic level, its magnetic moment and hence Zeeman splitting decreases. That may lead to a sub-linear *H*_t_(*T*) dependence.

## Conclusions

Dy_8_Mg_2_(SiO_4_)_6_O_2_ has an apatite type crystal structure in which the Dy atoms fully occupy the M2 sites and share the M1 sites with the Mg atoms. The coordination polyhedrons of Dy^3+^ are the same as in sister compound Dy_8_Ca_2_(SiO_4_)_6_O_2_, but with certain interatomic distances reduced. This provides the higher anisotropy of the crystal field and the magnetization. The modeling of electronic structure and the fitting of the temperature dependence of magnetic susceptibility show that Dy^3+^ has a strong easy axis magnetic anisotropy both for the M1 and M2 sites, similar to that in the sister compound. The magnetization relaxation of Dy_8_Mg_2_(SiO_4_)_6_O_2_ has been studied in detail over extended ranges of magnetic field and temperature. The sample exhibits a dual type magnetization relaxation with widely varying contributions from fast and slow relaxation paths. The latter path is characterized by relaxation times weakly dependent on the field and temperature. This path maintains large *τ* (a few seconds) at temperatures as high as 40 K and under a strong magnetic field of 50 kOe. It is concluded that commonly considered relaxation mechanisms – direct, Raman, and Orbach processes – do not appreciably contribute to the rate of SR. Taking into account the estimated electronic structure of Dy^3+^, it is proposed that the Orbach process, which may take place by the excitation to the 5th exited KD, is suppressed. The comparison with the Dy-doped phosphate apatites suggests that the Raman process is also hindered. In the model of dual relaxation^17^ the effective threshold field at which FR turns to SR is found to be proportional to *T*^2^. This may signify existence of a fine electronic structure formed due to inter-ion interactions and characterized by the gradual decrease of magnetic moment of the electronic level with the increase of its energy.

## Conflicts of interest

There are no conflicts to declare.

## Supplementary Material

RA-010-D0RA07069F-s001

RA-010-D0RA07069F-s002
