# Supplementary material for: Dysprosium magnesium silicate apatite featuring field and temperature stable slow magnetization relaxation
Source: RSC Adv. 2020 Oct 14;10(62):37588–95. doi: 10.1039/d0ra07069f (PMC9057236; doi:10.1039/d0ra07069f)
Supplement: RA-010-D0RA07069F-s001 [file RA-010-D0RA07069F-s001.pdf]

Electronic Supplementary Material (ESI) for RSC Advances.
This journal is © The Royal Society of Chemistry 2020

Electronic supplementary information

Table S1. Crystal structure refinement data for dysprosium magnesium silicate apatite.

Temperature (K)	293 K
Wavelength (Å)	1.5406
Formula	$Dy_8Mg_2(SiO_4)_6O_2$
Number of reflections	283
Number of data points	16749
Space group	$P6_3/m$ (176)
a (Å)	9.3081(2)
c (Å)	6.6742(2)
V (Å^3)	500.79(2)
Z	1
2θ range (deg.)	10 – 120
R_{wp}	0.010
R_{all}	0.029
ΔF_{max}, ΔF_{min} (e Å^{-3})	0.84, -1.28

Table S2. Atomic parameters and thermal displacement parameters (Å^2).

Atom	Dy1, Mg1	Dy2, Mg2	Si	O1	O2	O3	O4
Site	4f	6h	6h	6h	6h	12i	4e
SOF	0.542(3), 0.458(3)	0.972(3), 0.028(3)	1	1	1	1	1
x	1/3	0.0070(3)	0.4044(7)	0.3268(13)	0.6053(14)	0.3356(7)	0
y	2/3	0.23807(17)	0.3774(8)	0.4988(13)	0.4744(11)	0.2499(7)	0
z	-0.0058(8)	1/4	1/4	1/4	1/4	0.0665(8)	0.25
U_{iso}	0.0088(6)	0.0088(6)	0.014(2)	0.023(2)	0.023(2)	0.023(2)	0.023(2)

Table S3. Selected interatomic distances (Å) and angles (degrees). M1 - Dy1,Mg1; M2 - Dy2,Mg2

M1-O1	2.294(10)	3x	M1-M2	4.036(4)	3x
M1-O2	2.336(10)	3x	M2-M2	3.783(5)	2x
M1-O3	2.806(7)	3x	M2-M2	3.988(2)	4x
M2-O1	2.743(9)		Si-O1	1.617(17)	
M2-O2	2.376(19)		Si-O2	1.620(13)	
M2-O3	2.271(5)	2x	Si-O3	1.600(7)	2x
M2-O3	2.416(6)	2x	O1-Si-O2	113.9(6)	
M2-O4	2.184(2)		O1-Si-O3	111.4(5)	2x
M1-M1	3.260(8)		O2-Si-O3	109.6(5)	2x
M1-M1	3.414(8)		O3-Si-O3	99.9(4)	
M1-M2	3.992(4)	3x			

Table S4. Crystal field parameters (in Wybourne notation) derived in the program CONCORD for Dy^{3+} using experimental atomic coordinates of the coordination polyhedrons of Dy1 and Dy2 sites (from Table S2). Partial charges on the silicate oxygen atoms and on the intrachannel oxygen atom are -0.5 and -1.0 respectively (adjusted to fit the $\chi T(T)$ dependence).

Parameter	Value (cm^{-1})		
	Dy1<br>($a \rightarrow x$, $c \rightarrow z$)	Dy2<br>($a \rightarrow x$, $c \rightarrow z$)	Dy2<br>(z axis along easy magnetization axis)
B_{20}	865.30001	-456.44591	1039.1804
B_{22}		-478.43606	51.5511
B_{40}	-180.2481	172.04854	300.12362
B_{42}		10.38332	-48.96352
B_{43}	-49.13574		
B_{44}		-130.44089	19.8849
B_{60}	-24.02865	-15.41863	46.54823
B_{62}		-24.56548	-8.69366
B_{63}	-10.13758		
B_{64}		5.63719	6.01144
B_{66}	2.33517	15.97519	9.38259
B_{21}			
B_{41}			
B_{61}			
B_{65}			
B_{21}'			-86.03566
B_{22}'		-465.74269	
B_{41}'			129.0388
B_{42}'		157.53609	
B_{43}'	72.82336		-47.05804
B_{44}'		99.76525	
B_{61}'			-0.97924
B_{62}'		-15.08611	
B_{63}'	21.04695		3.06509
B_{64}'		-9.50989	
B_{65}'			-9.93864
B_{66}'	8.89063	-8.05367	

Table S5. Modeling with the program PHI using crystal field parameters listed in Table S4. The KDs of the ground multiplet $^6H_{15/2}$ are shown only.

Dy1

Energy (cm^{-1})	M_J (%)	g_z	g_x	g_y	Probability $M_J \rightarrow -M_J$
0	15/2 (99.98)	19.9987	0	0	
88.8457	13/2 (99.89)	17.2928	0.0001	0.0001	0.1482E-07
174.594	11/2 (99.74)	14.5899	0.0001	0.0001	0.1025E-05
255.924	9/2 (99.56)	11.8941	0	0	0.2374E-05
329.525	7/2 (99.35)	9.2140	0.1786	0.1786	0.6794E-02
390.907	5/2 (98.85)	6.5575	0.1867	0.1867	0.4781E-01
435.255	3/2 (68.38)	3.9753	0	0	0.1134E+01
458.574	1/2 (85.41)	10.6476	1.3062	10.6476	0.1451E+02

Dy2

Energy (cm^{-1})	M_J (%)	g_z	g_x	g_y	Probability $M_J \rightarrow -M_J$
0	15/2 (100)	19.9995	0	0	
173.782	13/2 (99.65)	17.2847	0.0001	0.0001	0.2161E+00
309.937	11/2 (99.80)	14.6404	0.0018	0.0021	0.8260E-06
409.580	9/2 (98.33)	12.0442	0.0022	0.0060	0.4562E+00
477.071	7/2 (95.27)	9.2474	0.7490	1.0345	0.7807E-01
516.887	5/2 (71.48)<br>1/2 (18.71)	7.3335	4.4427	6.8338	0.8383E+01
550.317	3/2 (58.46)<br>-1/2 (15.55)<br>-5/2 (22.67)	13.8204	1.0457	2.1845	0.1680E+02
586.637	1/2 (65.00)<br>-3/2 (29.68)	18.7428	0.0527	0.1175	0.3001E+02

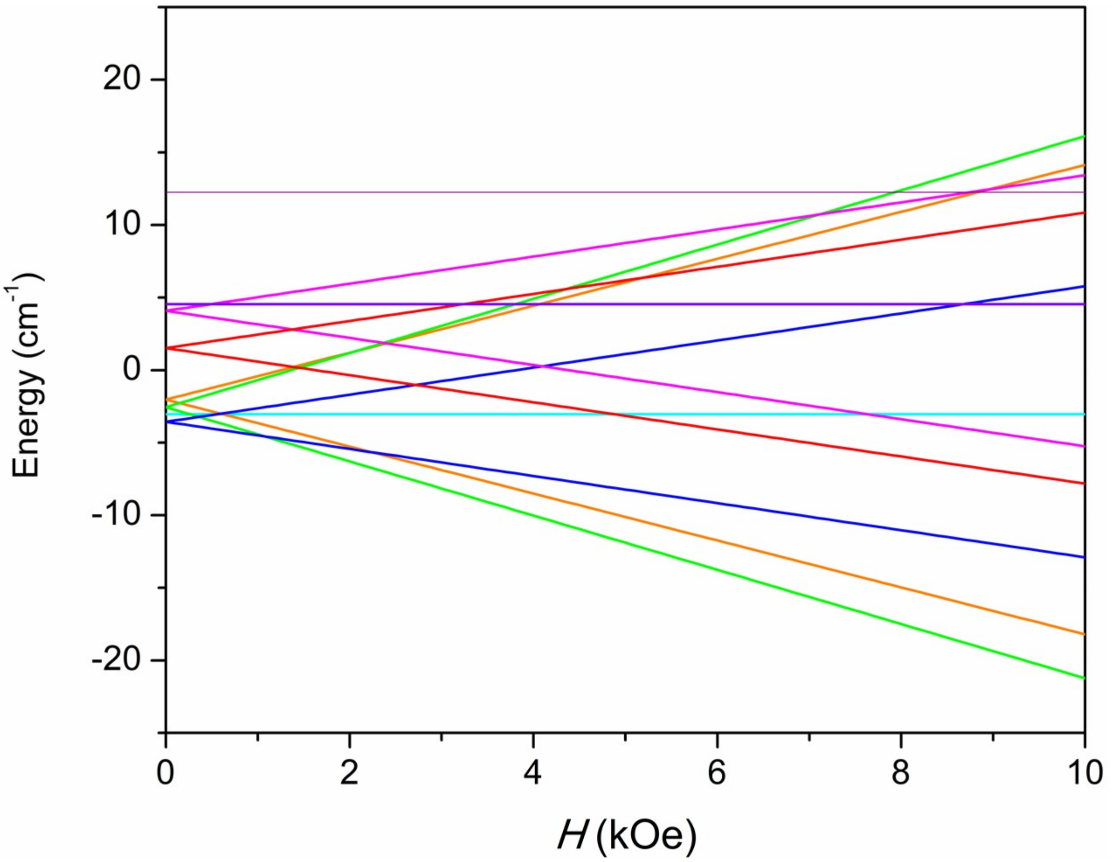

Fig. S1. Modeled energy diagram (KDs and singlets) for the $(Dy2)_6$ cluster and the level splitting in magnetic field along the easy magnetization axis of the cluster. The calculations were based on the experimental atomic positions taking into consideration dipole interactions only.

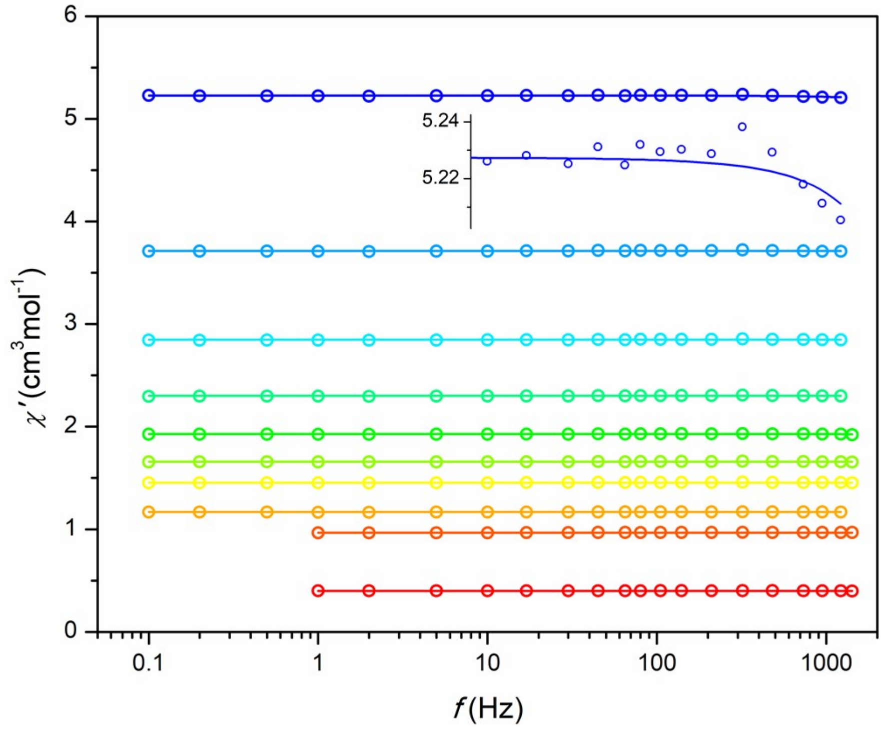

(a)

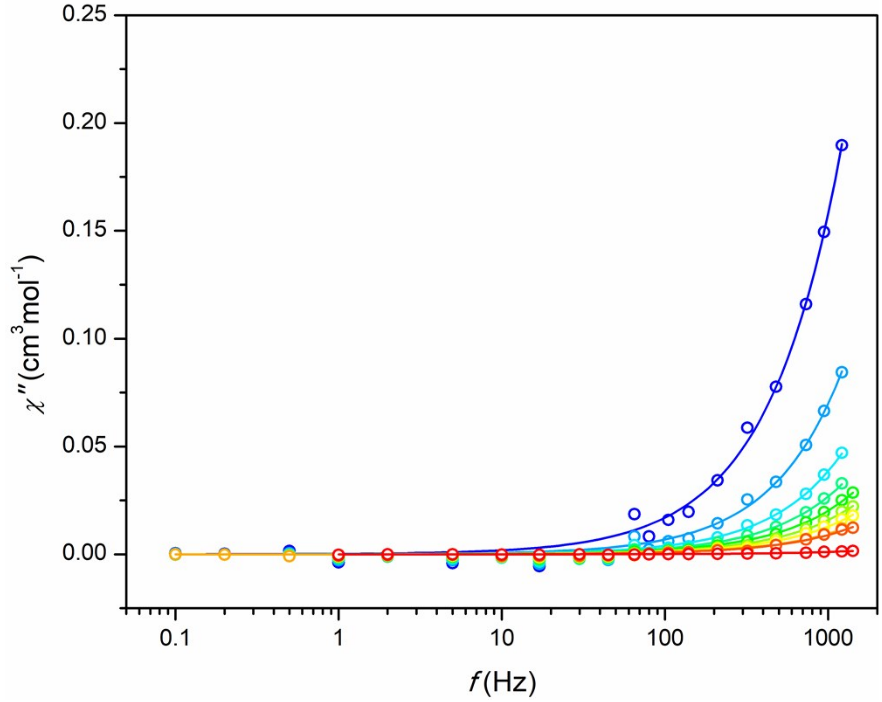

(b)

Figure S2. Frequency dependence of ac susceptibility per mol of Dy for $Dy_8Mg_2(SiO_4)_6O_2$ at different temperatures under a zero field. (a) – in-phase susceptibility χ', (b) – out-of-phase susceptibility χ''. Symbols – experimental points, lines – fitting. Color designation: blue – green – yellow – red, T = 2 – 8 K (step 1 K), T = 10, 12, 30 K, respectively.

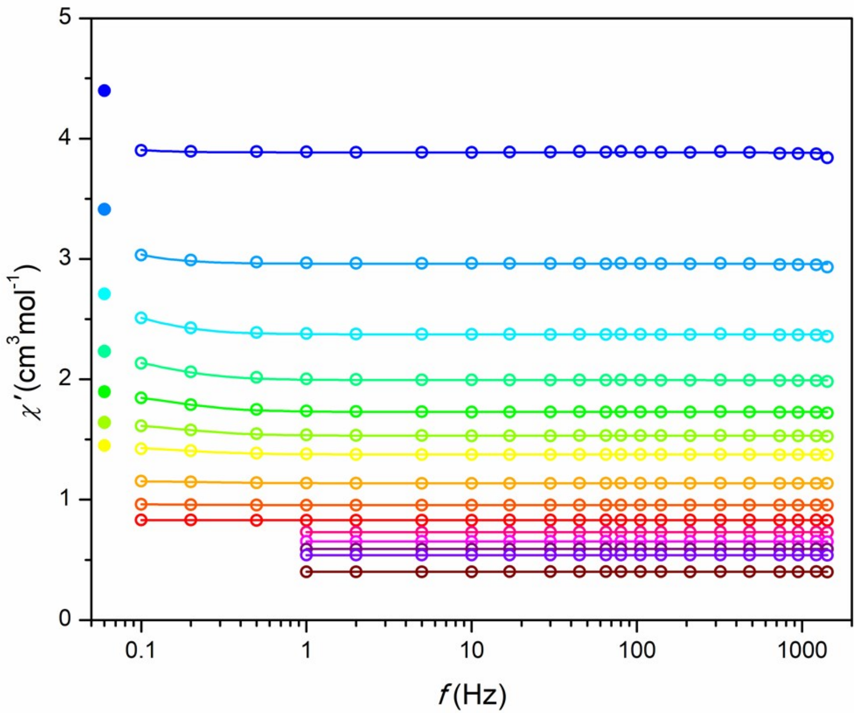

(a)

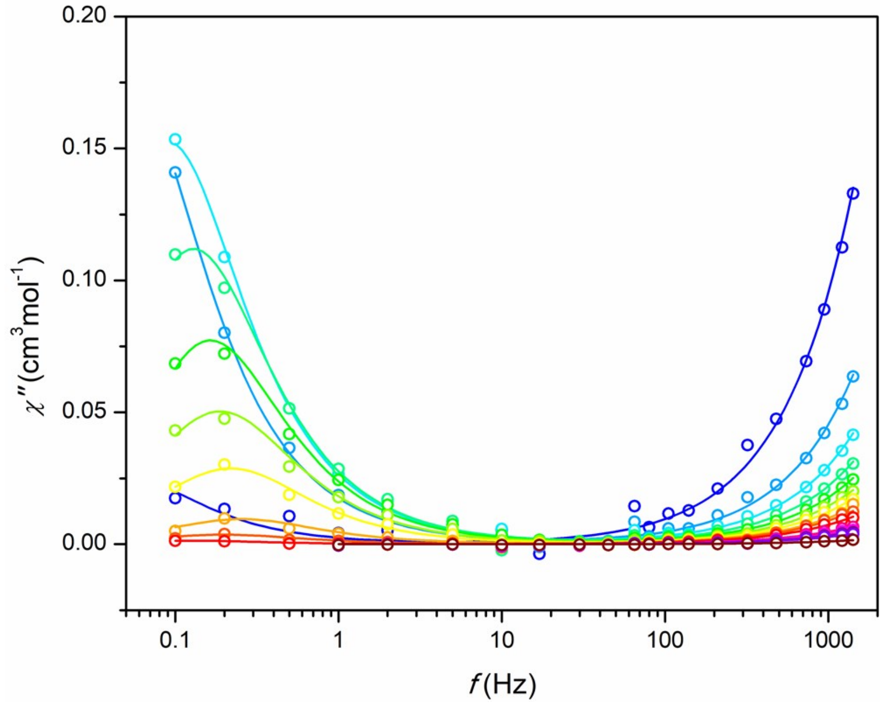

(b)

Figure S3. Frequency dependence of ac susceptibility per mol of Dy for $Dy_8Mg_2(SiO_4)_6O_2$ at different temperatures under a field of 1.5 kOe. (a) – in-phase susceptibility χ', (b) – out-of-phase susceptibility χ''. Open circles – experimental points, lines – fitting. Full circles on the left-side in (a) – experimental values of differential dc susceptibility under a field of 1.5 kOe (equal to χ_0). Color designation: blue – green – yellow – magenta – violet – wine, T = 2 – 8 K (step 1 K), T = 10 – 22 K (step 2 K), T = 30 K, respectively.

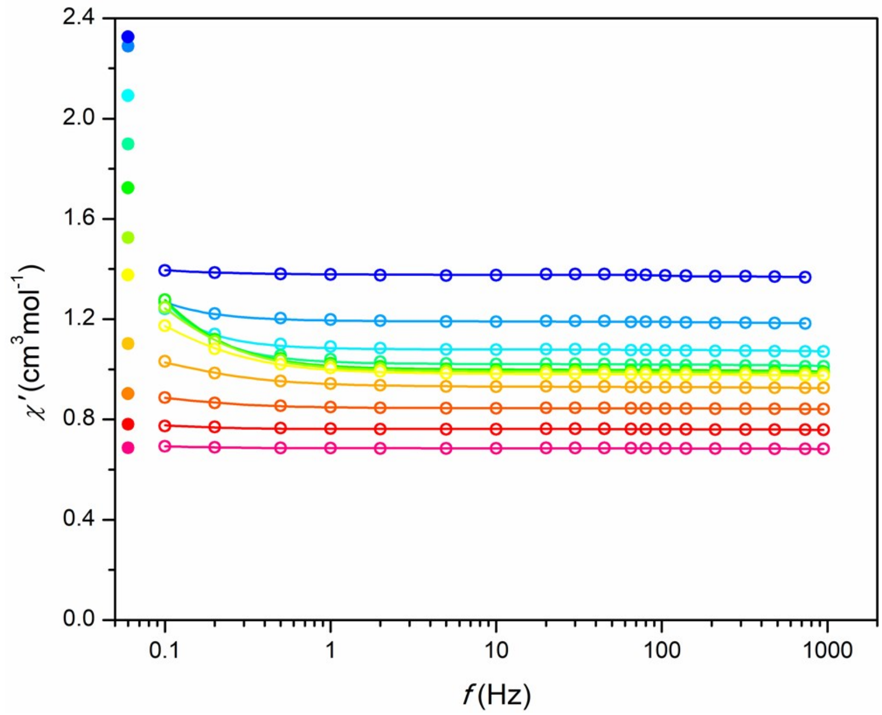

(a)

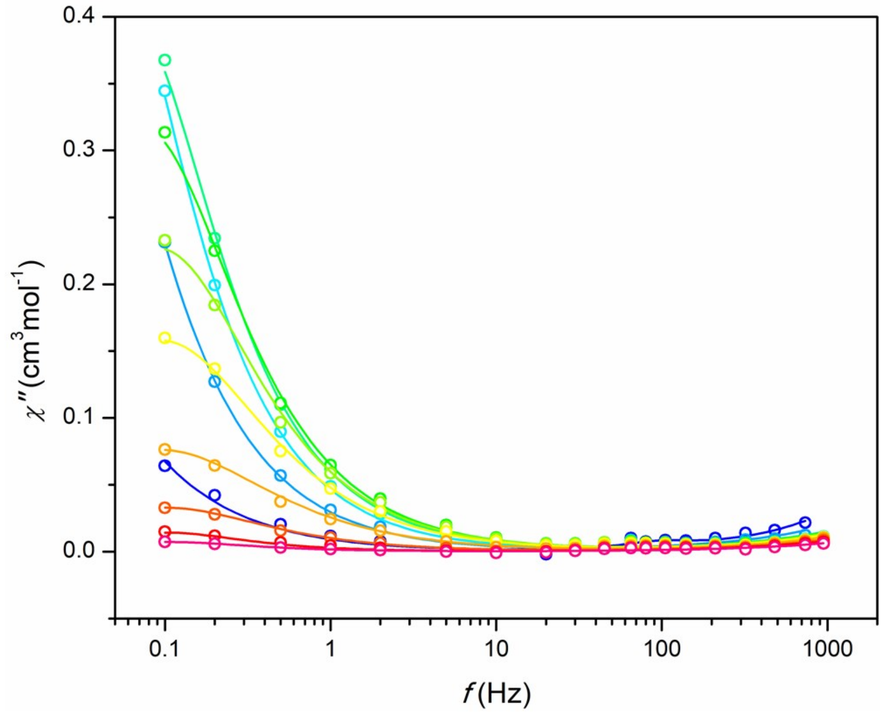

(b)

Figure S4. Frequency dependence of ac susceptibility per mol of Dy for $Dy_8Mg_2(SiO_4)_6O_2$ at different temperatures under a field of 4 kOe. (a) – in-phase susceptibility χ', (b) – out-of-phase susceptibility χ''. Open circles – experimental points, lines – fitting. Full circles on the left-side in (a) – experimental values of differential dc susceptibility under a field of 4 kOe (equal to χ_0). Color designation: blue – green – yellow – magenta, T = 2 – 8 K (step 1 K), T = 10 – 20 K (step 2 K), respectively.

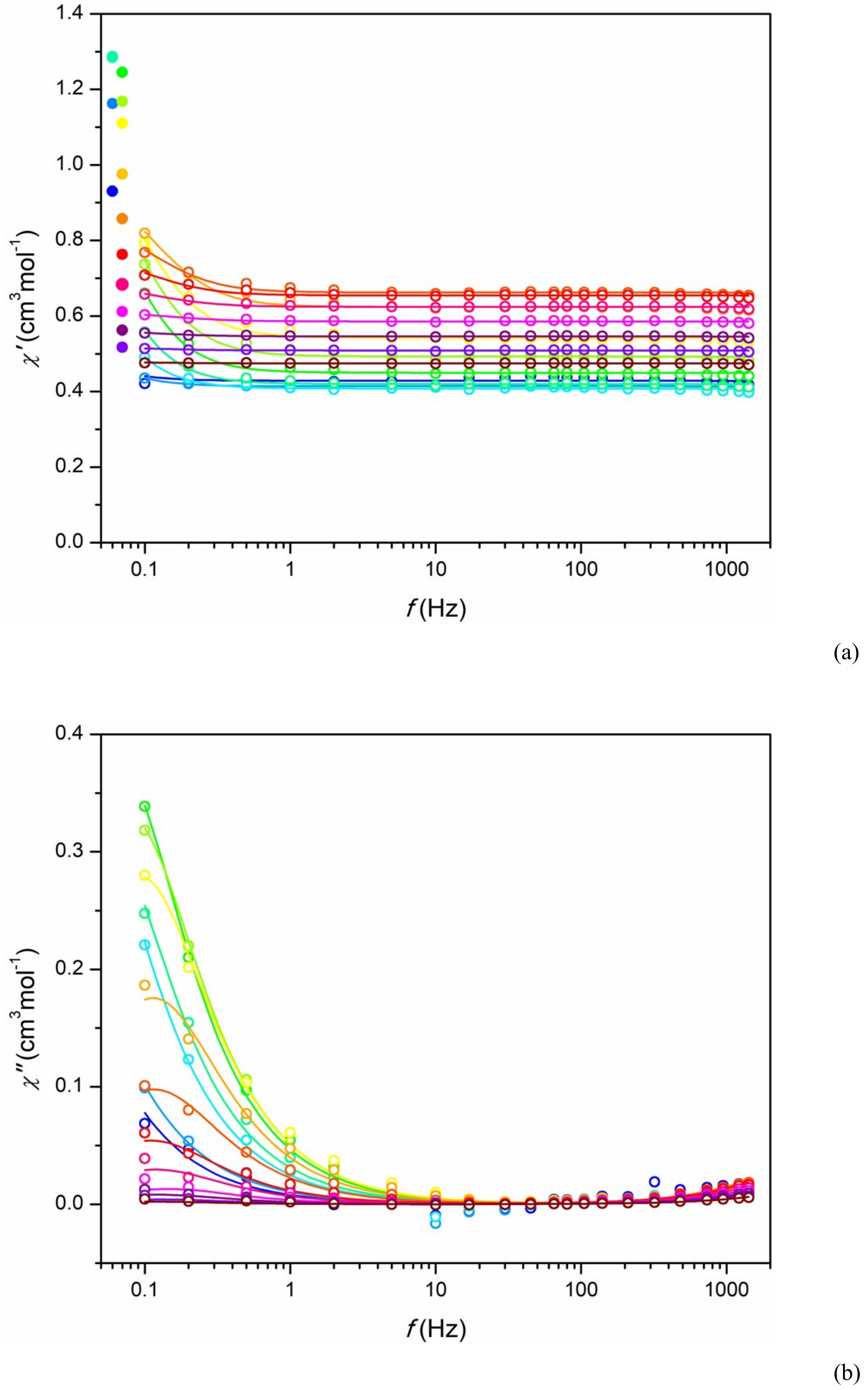

Figure S5. Frequency dependence of ac susceptibility per mol of Dy for $Dy_8Mg_2(SiO_4)_6O_2$ at different temperatures under a field of 8 kOe. (a) – in-phase susceptibility χ', (b) – out-of-phase susceptibility χ''. Open circles – experimental points, lines – fitting. Full circles on the left-side in (a) – experimental values of differential dc susceptibility under a field of 8 kOe (equal to χ_0). Color designation: blue – green – yellow – magenta – violet – wine, T = 2 – 8 K (step 1 K), T = 10 – 24 K (step 2 K), respectively.

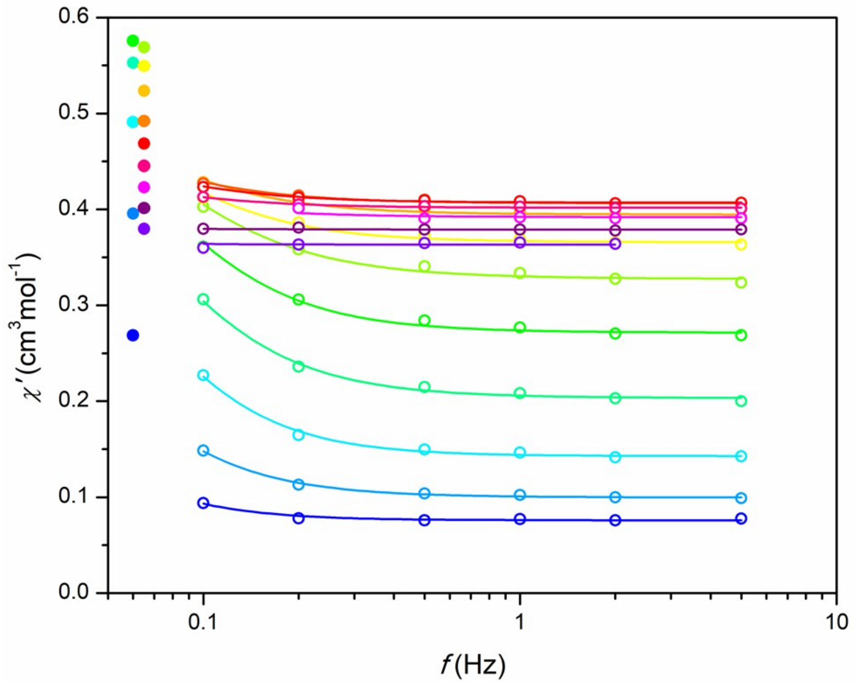

(a)

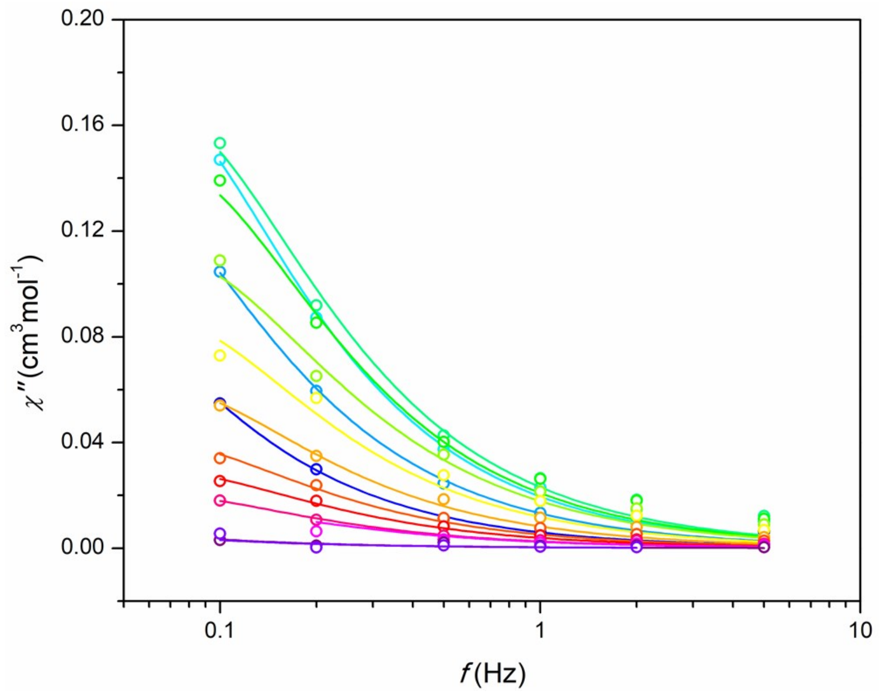

(b)

Figure S6. Frequency dependence of ac susceptibility per mol of Dy for $Dy_8Mg_2(SiO_4)_6O_2$ at different temperatures under a field of 20 kOe. (a) – in-phase susceptibility χ', (b) – out-of-phase susceptibility χ''. Open circles – experimental points, lines – fitting. Full circles on the left-side in (a) – experimental values of differential dc susceptibility under a field of 20 kOe (equal to χ_0). Color designation: blue – green – yellow – magenta – violet, T = 4 – 30 K (step 2 K), respectively.

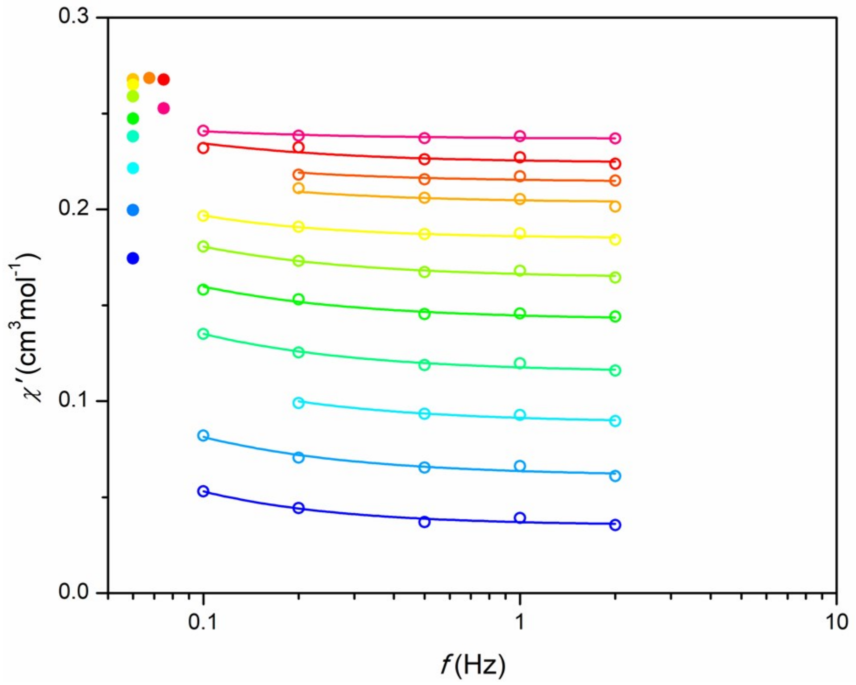

(a)

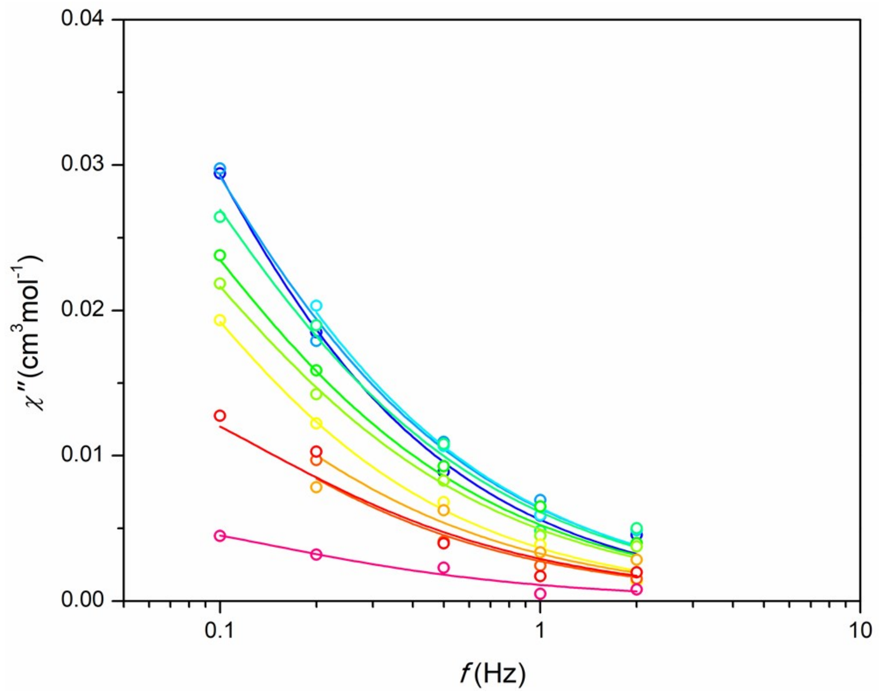

(b)

Figure S7. Frequency dependence of ac susceptibility per mol of Dy for $Dy_8Mg_2(SiO_4)_6O_2$ at different temperatures under a field of 50 kOe. (a) – in-phase susceptibility χ', (b) – out-of-phase susceptibility χ''. Open circles – experimental points, lines – fitting. Full circles on the left-side in (a) – experimental values of differential dc susceptibility under a field of 50 kOe (equal to χ_0). Color designation: blue – green – yellow – rose, T = 12 – 30 K (step 2 K), T = 40 K, respectively.

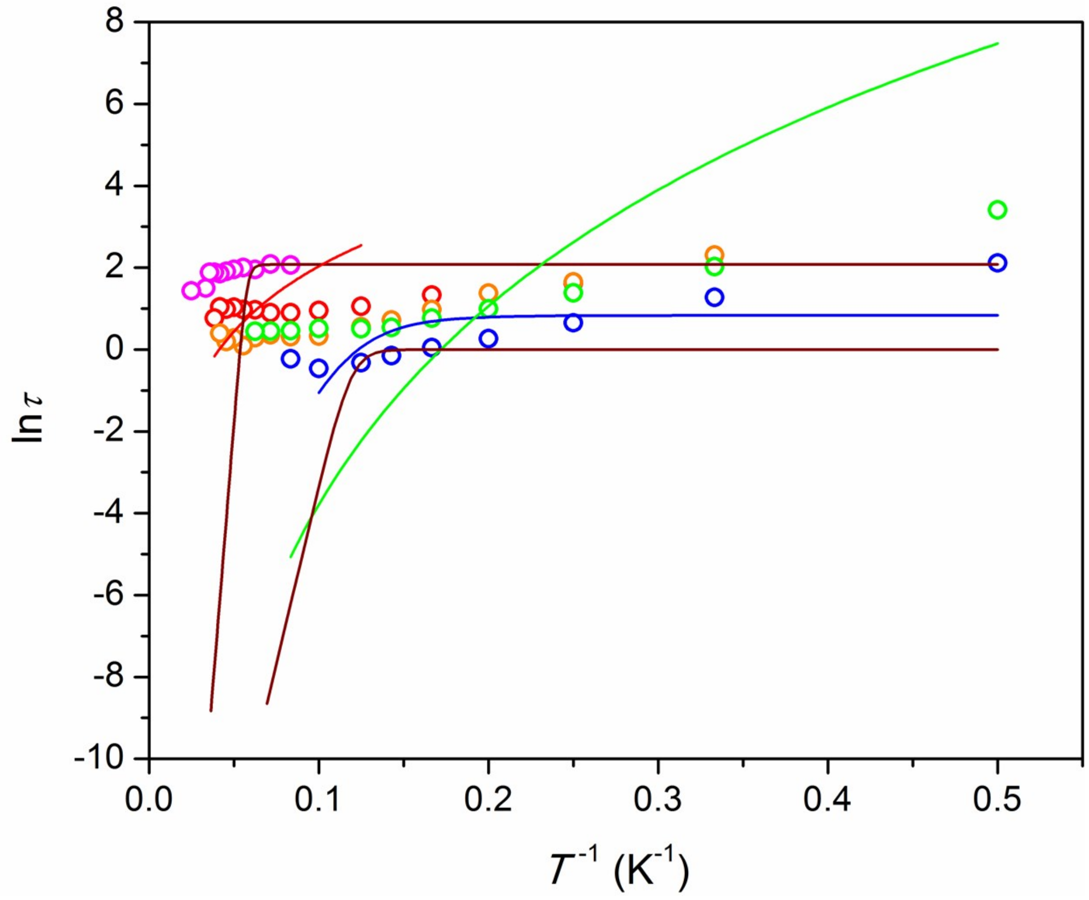

Figure S8. Temperature dependence of $\ln\tau$ for the SR path in the Dy-Mg silicate apatite. Experimental points – symbols; blue, green, orange, red, and magenta – under a field of 1.5, 4, 8, 20, and 50 kOe, respectively. Lines – modeling and fitting. Brown lines – modeling of the relaxation taking into consideration the Orbach and QTM mechanisms: $1/\tau = 1/\tau_{QTM} + (1/\tau_0)\exp(-U_{eff}/kT)$; upper line – U_{eff} = 517 cm^{-1} (calculated energy of the 5th exited KD for the Dy2 site), $\tau_0 = 10^{-12}$ s, τ_{QTM} = 8 s; lower line - U_{eff} = 174 cm^{-1} (calculated energy of the first exited KD for the Dy2 site), $\tau_0 = 10^{-9}$ s, τ_{QTM} = 1 s. Green and red lines – fitting the relaxation time using the Raman mechanism for the data under a field of 1.5 and 20 kOe respectively: $1/\tau = cT^n$; green line – n = 7 (low temperature region), $c = 4.4\cdot10^{-6}$ s^{-1} K^{-n}; red line – n = 2.3 (high temperature region), $c = 6.6\cdot10^{-4}$ s^{-1} K^{-n}. The Raman mechanism is considered as a Raman I. A field dependent Raman II is discarded since, according to this mechanism, τ has to decrease fast with the increasing magnetic field in a high field region, whereas, in the experiment, τ gradually grows with the increasing field.
